# Public Sanitation

**Published:** 1920-07-10

**Authors:** 


**THE PUBLIC WELL-BEING—(*continued*)**

### Public Sanitation.

At their annual conference at Sheffield the members of the Institute of Cleansing Superintendents discussed a matter of supreme importance to the public health. Even in these days, as the Lord Mayor of Sheffield pointed out, people do not generally realise the importance of the work done by the cleansing departments of our municipalities, and this lack of appreciation, it may be added, is sometimes as evident among members of local authorities as it is among the general public. A

**THE PUBLIC WELL-BEING—(*continued*).**

modern development in public sanitation is the tendency to depart from the old custom of destroying all refuse by burning in favour of a system which utilises waste products as far as possible for commercial purposes. The task of keeping our cities and towns clean is certainly accomplished in this country with more skill and scrupulous care than in large parts of France and in several other European countries—a fact which members of the demobilised British Army who saw active service on the Continent during the late war would be prompt to acknowledge. But a great deal remains to be done if the utmost resources of modern civilisation are to be exploited fully, and Mr. J. A. Priestley, the new President of the Institute, was right in calling attention to the need for a greater uniformity in methods, appliances, and records in cleansing work.

He went on to suggest the formation of a Technical Committee, to which many of these matters could be referred, and whose findings should be put into practice. Such a Committee, possessing national or Local Government support, and with power and means to carry out practical experiments, tests, and investigations, would probably be a most useful help to all local authorities. The question of uniformity in the disposal of refuse will have to be tackled with determination sooner or later. In the matter of storage one finds "fixed ashpits, open and enclosed, portable receptacles, wooden, galvanised, nondescripts, with lids and without, under cover and in the open, on stands or just anywhere, and of any or every size, shape, and condition." As for removal, there are daily, bi-weekly, weekly, and fortnightly collections. Bins are placed on the kerb by occupiers or fetched out by the scavengers, emptied on the street, or into skips, or direct into the cart. With regard to transport there is a very "babel of confusion." Horsed carts and wagons—open, closed, with loose covers, swing lids, sliding doors, fixed bodies, tipping bodies—motors—electric, petrol, steam, open, covered, large, small—tipping gear of every variety, vehicles of endless variety in size, make, and appearance, from perambulators to furniture-vans. That is Mr. Priestley's description, and it is a true one. We agree that a few well-defined types would meet the case, and that it is both inefficient and unnecessary to have on the one hand the horsed vehicle without tipping body carting refuse three miles from the point of collection to a tip, and, on the other hand, the mechanical pantechnicon collecting light refuse in half-mile circles.

### Lines of Reform.

Even of more importance is the question of disposal. We have already indicated that the old methods are realised to be out of date and by no means friendly to the public health. Tipping—a method which is a prolific breeder of disease and infection—or destruction have for two generations been the alternative methods of refuse disposal. But both have been found wanting, and we believe the time is coming when both these methods will be exchanged largely by the process known as utilisation. Vast quantities of trade refuse which is at present destroyed could be transformed into useful commercial purposes, and particularly in regard to waste paper, which makes up a very large proportion of common refuse. When once a practical way of separating the dirt from the paper has been devised the problem of re-pulping the paper on a commercial basis may be solved, as was suggested, by the local authorities pooling their resources and despatching the baled material to a common centre, where it could be cleaned, re-pulped, and supplied as pulp-boards to the paper-maker. The disposal of tins, always a fruitful source of foulness, the cheapest method appears to be that of burning off the tin and baling them in hydraulically operated presses for sale as scrap metal. According to Mr. W. Mortimore, of Manchester, however, a suitable plant for the recovery of the tin has not yet appeared on the market for sale to municipalities, although private firms have plants which are reported to be remunerative. The whole question of utilisation is, indeed, one of very great interest, and may before long revolutionise the present system of refuse disposal.

### A Deadly Practice.

One subject which might very well have engaged the attention of the cleansing superintendents in their debates is that of street sweeping at night. It is the disgusting habit of the cleansing departments in most of the large English towns to send out about midnight a procession of horse-drawn rotary brushes to sweep the streets of the dust or mud that has accumulated during the day. No great harm results after a rainy day. But in the heat and drought of the summer this grossly unhygienic practice is fraught with serious danger. Half the dust and filth churned up by these heavy and powerful brushes is sent house-high in evil-smelling pillars of thick fog, which contain more billions of microbes than the human imagination can encompass. Having sprayed the houses and soiled the windows of every shop and private dwelling in its way, and enveloped in its embrace any unlucky passer-by, the pillar of dust slowly descends again, and leaves the street dirtier and more offensive and more disease-bearing than it was before. The remedy is so simple that to the plain man it is an amazement that all municipalities have not adopted it. A few years ago the writer was in Cologne, and there the streets were swept in a decent and sanitary manner by attaching to the rotary brush a water-sprayer, which laid the dust at each revolution. Here is a chance for some enterprising English city to learn a lesson in cleanliness from our late enemy.

